# Binary acoustic metasurfaces for dynamic focusing of transcranial ultrasound

**DOI:** 10.3389/fnins.2022.984953

**Published:** 2022-09-01

**Authors:** Zhongtao Hu, Yaoheng Yang, Lu Xu, Yao Hao, Hong Chen

**Affiliations:** ^1^Department of Biomedical Engineering, Washington University in St. Louis, Saint Louis, MO, United States; ^2^Department of Radiation Oncology, Washington University School of Medicine, Saint Louis, MO, United States

**Keywords:** transcranial focused ultrasound, acoustic lens, dynamic focusing, beam steering, neuromodulation, binary acoustic metasurface, FUS-BBBD, aberration correction

## Abstract

Transcranial focused ultrasound (tFUS) is a promising technique for non-invasive and spatially targeted neuromodulation and treatment of brain diseases. Acoustic lenses were designed to correct the skull-induced beam aberration, but these designs could only generate static focused ultrasound beams inside the brain. Here, we designed and 3D printed binary acoustic metasurfaces (BAMs) for skull aberration correction and dynamic ultrasound beam focusing. BAMs were designed by binarizing the phase distribution at the surface of the metasurfaces. The phase distribution was calculated based on time reversal to correct the skull-induced phase aberration. The binarization enabled the ultrasound beam to be dynamically steered along wave propagation direction by adjusting the operation frequency of the incident ultrasound wave. The designed BAMs were manufactured by 3D printing with two coding bits, a polylactic acid unit for bit “1” and a water unit for bit “0.” BAMs for single- and multi-point focusing through the human skull were designed, 3D printed, and validated numerically and experimentally. The proposed BAMs with subwavelength scale in thickness are simple to design, easy to fabric, and capable of correcting skull aberration and achieving dynamic beam steering.

## Introduction

Transcranial focused ultrasound (tFUS) provides a platform technology for various brain applications. It can non-invasively penetrate through the skull bone and focus on targeted brain locations with millimeter precision. tFUS at low intensity levels has been used for neuromodulation by targeting different parts of the brain (Legon et al., 2014; Ibsen et al., 2015; Qiu et al., 2020; Yang et al., 2021; Darmani et al., 2022). tFUS combined with microbubbles can non-invasively, locally, and reversibly disrupt the blood-brain barrier for brain drug delivery (Chen and Konofagou, 2014; Lipsman et al., 2018; Chen et al., 2021; Hu et al., 2022a) or brain tumor-derived molecular biomarker release (Zhu et al., 2018; Meng et al., 2021; Pacia et al., 2022). High-intensity tFUS has been used in the clinic for the treatment of essential tremor and Parkinson’s disease by thermally ablating the diseased brain sites (McDannold et al., 2010; Coluccia et al., 2014; Elias et al., 2016). tFUS with high pressure amplitudes and short pulses can be used for histotripsy by generating cavitation bubble clouds to mechanically fractionate brain tissue at the targeted site (Vlaisavljevich et al., 2014). Achieving high spatially precision of tFUS targeting is critical to all these applications in the brain. However, the high acoustic impedance and complex poroelastic and varying thickness of the skull generate strong scattering, refraction, and attenuation on the propagating ultrasonic waves, resulting in ultrasound beam aberrations and defocusing (Ammi et al., 2008; Pichardo et al., 2011; Hu et al., 2019; Montanaro et al., 2021).

Several tFUS technologies have been developed to overcome the skull-induced beam aberration. The most commonly used approach is to utilize phased arrays, which focus the ultrasound beam at the desired target by modulating the phase delay of each independent transducer element (Deng et al., 2020; Sukovich et al., 2020; Adams et al., 2021; Bawiec et al., 2021; Leung et al., 2021). Although phased arrays have the advantage of being dynamically programmable, these arrays need a large number of transducer elements and complex electronics (Melde et al., 2016). Another promising approach is to place an acoustic lens in front of a single-element transducer to correct the skull-induced beam aberration. The thickness profile of the acoustic lens is derived from the phase profile obtained by numerical simulation of transcranial wave propagation from the targeted point (Tian et al., 2017; Zhang et al., 2018; Zhu and Assouar, 2019; Brown et al., 2020; Kim et al., 2021). The acoustic lens is then 3D printed, which provides a low-cost and easy-to-implement solution to compensate for the skull aberration.

Several acoustic lenses were designed for tFUS. Maimbourg et al. (2018) focused a 1 MHz ultrasound beam through a human skull with a single-element focused transducer coupled with a tailored silicone acoustic lens and demonstrated the possibility of adjusting the focus by physically moving the transducer with the lens (Maimbourg et al., 2020). Jiménez-Gambín et al. (2019, 2020), printed acoustic holographic lenses coupled with a single-element flat transducer for the formation of double foci beam, self-bending beam, volumetrically focused beam, and ultrasonics vortices inside the skull. Andrés et al. (2022) numerically studied the performance of an acoustic lens positioned at the temporal bone window for generating acoustic holograms at the thalamic nuclei. Although these existing studies demonstrated the capability of acoustic lenses, they have the common limitation that the generated ultrasound beams are static. Once the lenses are printed, they can only target one specific location without mechanically moving the transducers with the lenses. This shortcoming limits their flexibility in applications when targeting multiple brain locations are needed. For example, tFUS neuromodulation may need to target different parts of the brain to probe their response to ultrasound stimulation (Jones et al., 2022). Therapeutic applications of tFUS in the brain often require sonication of a large brain volume where multiple-point targeting is critical (Magnin et al., 2015).

The objective of this study was to develop a binary acoustic metasurface (BAM) lens to achieve double functionality: correcting the beam aberration induced by the skull and achieving dynamic focusing. We designed BAMs by binarizing the phase distribution at the surface of the metasurfaces. The phase distribution was calculated based on time reversal to correct the skull-induced phase aberration. The binarization enabled the ultrasound beam to be dynamically steered along wave propagation direction by adjusting the operating frequency of the incident ultrasound wave. We demonstrated the capability of BAMs in transcranial beam aberration correction and dynamic beam steering experimentally and numerically. We also showed that BAMs could be designed to form multi-foci, enabling simultaneous and dynamic targeting of multiple brain structures.

## Materials and methods

### Binary acoustic metasurface design and fabrication

**FIGURE 1:**
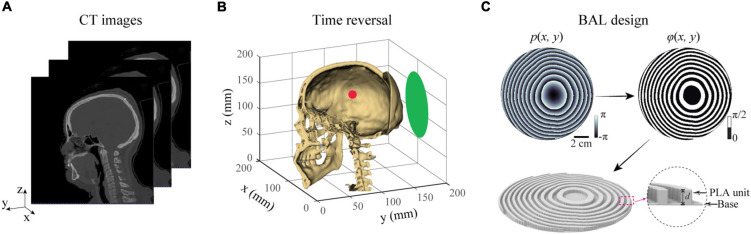
Methods for designing a BAM for transcranial focused ultrasound. **(A)** CT-scan of a human head was used to perform numerical simulations of the ultrasound wave propagation in the brain. **(B)** A virtual acoustic source (red dot) was placed at the targeted brain location. Time reversal was then used to calculate the phase profile on the surface of the lens (green plane). **(C)** A BAM was designed by binarizing the calculated phase profile from **(B)**, and rendered into a 3D-printed model with two coding bits, a polylactic acid (PLA) unit for bit “1” and a water unit for bit “0”.

BAMs were designed in three steps. First, we extracted the geometry and acoustic properties of a human skull from CT images, as illustrated in Figure 1A. Second, these properties were then used in numerical simulations by the time-reversal technique to calculate the acoustic wavefront generated by virtual point sources inside the skull (Figure 1B). The acoustic wavefront was then recorded on the surface of an acoustic lens located outside the skull and transformed into a phase profile. Third, a BAM was generated by binarizing the phase profile ranging from 0 to 2π to 0 and π/2 with φ(*x*,*y*) = 0 for *p*(*x*,*y*) > 0 and φ(*x*,*y*) = π/2 for *p*(*x*,*y*) < 0, and the binary phase map was rendered into a 3D-printed model, as shown in Figure 1C. The binary design of BAMs make it work at broadband frequency range and enables the dynamic focusing properties as reported in previous studies (Xie et al., 2017; Tang et al., 2021; Hu et al., 2022b), for it allows broadband frequency range transmitting the printing elements with the equal amplitude and phase shift. However, it is difficult for the acoustic lens designed with gradient phase to achieve this property. This method can be used to design BAMs for single-point focusing by placing a single virtual point source inside the skull. It can also be used to design BAMs for multi-point focusing by placing multiple virtual point sources inside the skull.

The designed BAM was then manufactured by 3D printing. The phase delay of each pixel on the BAM is proportional to the thickness of polylactic acid (Lin et al., 2015). To produce a phase delay of π/2, the thickness (*d*) of unit “1” was calculated using $\frac{2\,\pi \,f}{c_{1}}\,d-\frac{2\,\pi \,f}{c_{2}}\,d=\frac{\pi }{2}$, where *c*_*1*_ and *c*_*2*_ are the sound speed of the water and polylactic acid, respectively, and *f* is the operating frequency. Thus, the thickness is represented as *d* = *c*_1_*c*_2_/4*f*(*c*_2_−*c*_1_). The pressure transmission coefficient (*T*) of each unit “1” can be calculated using (Jiménez et al., 2017): $T=\frac{2\,Z_{r}}{2\,Z_{r}\,\cos\left(2\,\pi \,f\,d/c_{2}\right)-i\,\left(Z_{r}^{2}+1\right)\,\text{sin}\,\left(2\,\pi \,f\,d/c_{2}\right)}$, where the normalized acoustic impedance is given by *Z*_*r*_ = *Z*_2_/*Z*_1_, the impedance of water is given by *Z*_1_ = ρ_1_*c*_1_, and the impedance of polylactic acid is given by *Z*_2_ = ρ_2_*c*_2_. The terms ρ_1_ and ρ_2_ are the densities of water and polylactic acid, respectively. The acoustic properties of polylactic acid material are obtained experimentally using a pulse-echo technique in a cubic structure, resulting in a measured sound speed of 2,212 m/s, and a density of 1,223 kg/m^3^, and absorption of 3.54 dB/cm for 500 kHz. These measurements matched those reported in previous studies (Jiménez-Gambín et al., 2019; Tarrazó-Serrano et al., 2019). Water as the surrounding medium has a sound speed of 1,480 m/s and mass density of 1,000 kg/m^3^ at room temperature. The thickness of the BAM was calculated to be 2.35 mm for the operating frequency of 500 kHz, which was approximately 0.8λ (λ = 2.97 mm). The BAM consisted of two printed parts. The first part was the polylactic acid unit on the lens with a depth of 2.25 mm that provided π/2 phase shift for the 500 kHz transmitted ultrasound wave. The second portion was the base plate printed at a thickness of 0.10 mm, which was needed to stabilize the lens. The transmission coefficient was 99.6% for unit “1,” indicating efficient transmission through the lens. As a proof of concept, we designed and 3D printed BAMs for generating tFUS with a single focus or two foci.

### Numerical and experimental method

The 3D-printed BAMs were evaluated numerically and experimentally using an *ex vivo* human skullcap. The *ex vivo* human skullcap, which was dry from storage in air, was immersed in water and degassed for a minimum of 24 h in a vacuum chamber at -0.1 MPa measured by a pressure gauge (Nisshin 1.6, Nisshin Seifun Group Inc., Tokyo, Japan) to eliminate air bubbles trapped in the skull before use.

For the numerical studies, simulations were performed using an open-source MATLAB toolbox, k-Wave, a pseudospectral method with k-space dispersion correction (Treeby and Cox, 2010; Park et al., 2019; Wu et al., 2020; Hu et al., 2021). A graphics processing unit (Nvidia Tesla V100, Nvidia Corporation, Santa Clara, CA, United States) was used to accelerate the 3D simulations. The acoustic properties of the *ex vivo* human skullcap were obtained from CT scans using a clinical CT scanner (Siemens Somatom Confidence, Siemens Healthcare, Erlangen, Germany). The density and sound speed of the skull were converted from the Hounsfield units of the CT images by the function “hounsfield2density” in the k-Wave toolbox. This function uses a piecewise linear fit to the experimental data reported by Schneider and Mast (Schneider et al., 1996; Mast, 2000). The density data of the skull ranges between ρ_min_ = 1,000*kg*/m^3^, and ρ_max_ = 3,200*kg*/m^3^, with an average density of ρ_mean_ = 1,525*kg*/m^3^, and the sound speed of skull ranges between c_min_ = 1,480m/s and c_max_ = 4,050m/s, with an average sound speed of c_mean_ = 2,542m/s, matching those reported in the previous literature (Marsac et al., 2017). The attenuation was obtained by the power law model as proposed by Aubry et al. (2003) and Constans et al. (2017). The size of each CT image was 512 × 512 × 351 voxels with a spatial resolution of 0.3906 mm in the xy-plane and 0.6000 mm in the z-axis. After linear interpolation, a numerical grid with an isotropic spatial resolution of 0.2mm was generated. A numerical temporal step of △t = 20*ns* was used. The Courant-Friedrichs-Lewy number was 0.1 and the spatial sampling was approximately 15 grid points per wavelength in water at 500 kHz. These parameters were fixed for all simulations in this study.

**FIGURE 2:**
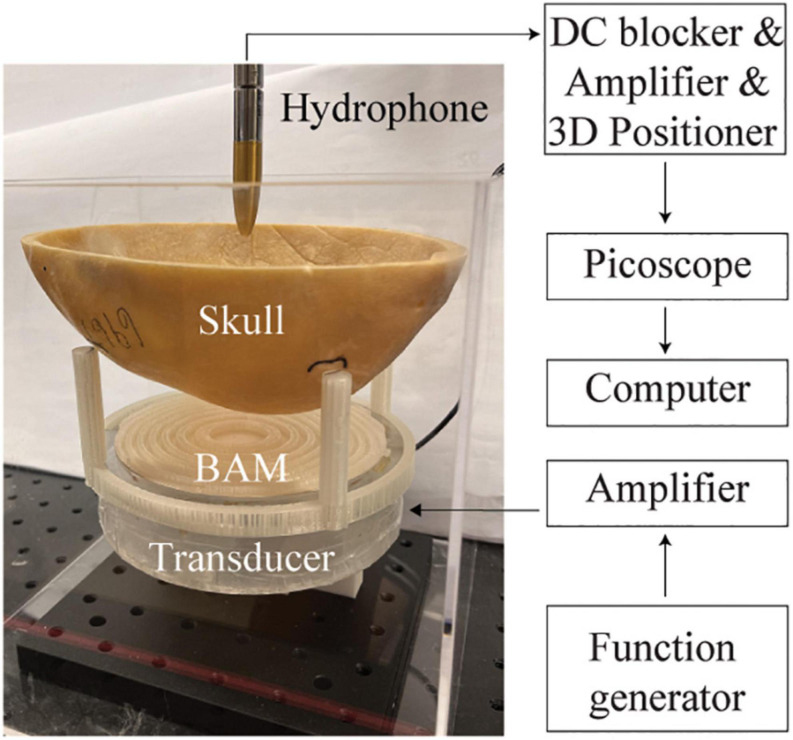
Experimental setup for validating the designed BAM coupled with a plat ultrasound transducer.

For the experimental studies, the 3D-printed BAMs were coupled with a flat transducer. The flat transducer had a frequency of 500 kHz and an aperture of 120 mm. It was made of a single-element circular lead zirconate titanate (PZT) ceramic (DL-20, Del Piezo Specialties LLC, West Palm Beach, FL, United States). Positive and negative electrodes of the PZT ceramic were soldered with wires that were connected with an electrical driving system composed of a function generator (Model 33500B, Keysight Technologies Inc., Englewood, CO, United States) and a power amplifier (1020L, Electronics and Innovation, Rochester, NY, United States). The transducer was encased in a 3D-printed housing. The experimental setup is shown in Figure 2. The ultrasound waves generated by the flat transducer passing through a BAM and then skull was measured by a hydrophone (HGL-200, ONDA Corporation, Sunnyvale, CA) in a water tank filled with degassed and deionized water. The hydrophone was connected to a pre-amplifier (AG-20X0, Onda Corp., Sunnyvale, CA, United States) and a digital oscilloscope (Picoscope 5443D, St. Neots, United Kingdom) and moved in 3D using a computer-controlled 3D stage (PK245-01AA, Velmex Inc., NY, United States).

## Results

### Binary acoustic metasurface for single focusing

**FIGURE 3:**
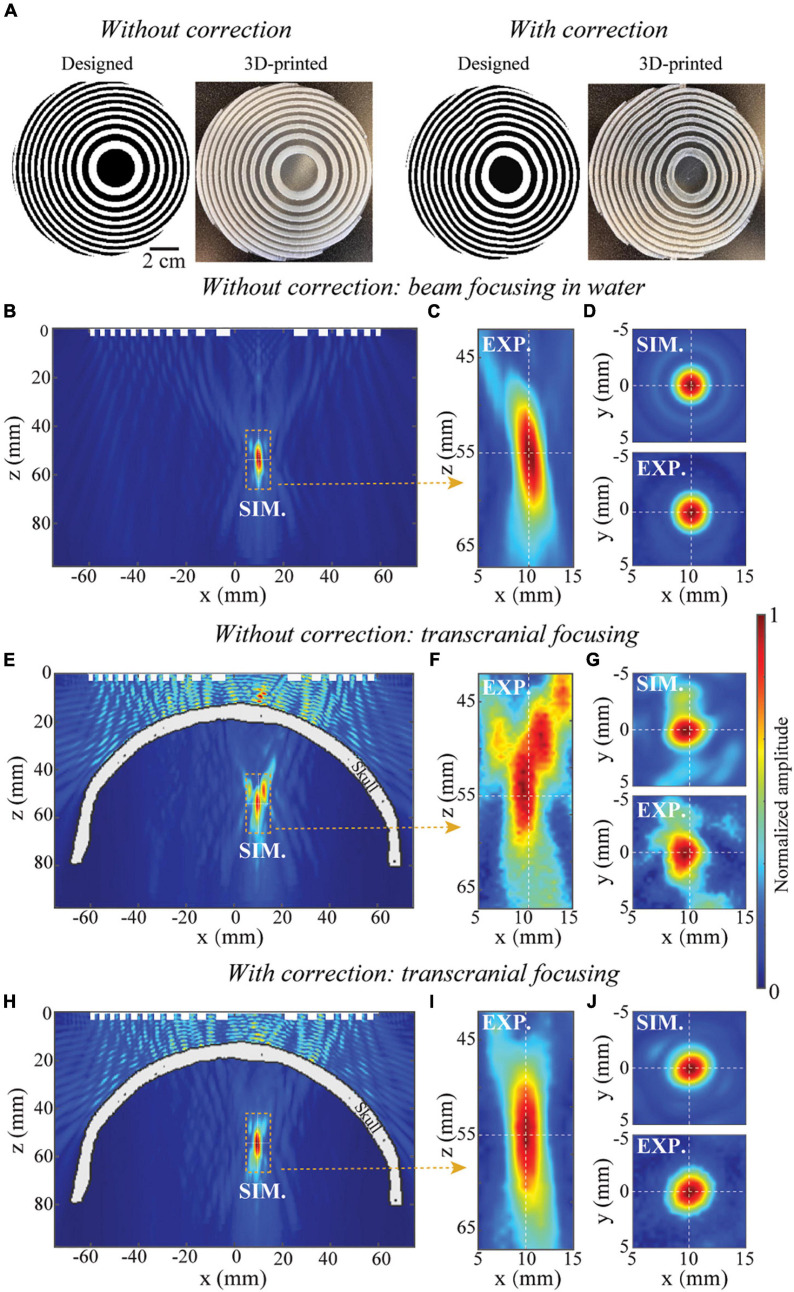
Single-focus BAM for tFUS. **(A)** The designed and 3D-printed BAMs without and with skull aberration correction, respectively. The white and black unit represent units “π/2” and “0”. **(B,C)** Simulated (SIM) and experimental (EXP.) calibration of pressure distribution along xz-plane (axial view) of beam focusing in homogenous media, **(D)** the corresponding simulated and experimental pressure distribution along xy-plane (transverse view). **(E,F)** Simulated and experimental calibration of pressure distribution along xz-plane of transcranial focusing using the lens without correction, **(G)** the corresponding simulated and experimental pressure distribution along xy-plane. **(H,I)** Simulated and experimental calibration of pressure distribution along xz-plane of transcranial focusing using the lens with correction, **(J)** the corresponding simulated and experimental pressure distribution along xy-plane. The pressure fields are normalized to the peak pressure.

We start with designing, manufacturing, and evaluating BAMs for generating a single focus. The simulated and measured acoustic pressure fields generated by single-focus BAMs are shown in Figure 3. Figure 3A shows the designed and 3D-printed BAMs without and with skull aberration correction at (x=10,*y* = 0,*z* = 55)mm, respectively. Here, the BAM designed without correction was taken as a reference. The pressure field in the xz-plane (y = 0 mm) and xy-plan (z = 55 mm) generated by the BAM in water without skull is shown in Figures 3B–D. The respective full-length half maximum (FLHM) and full-width half maximum (FWHM) from the simulation were 13.1 and 3.3 mm, and the corresponding experimental measurements were 14.6 and 3.6 mm. The ultrasound field generated by the BAM without phase correction for transcranial focusing is shown in Figures 3E–G. Several side lobes appear around the main beam in both simulation and experiment along the xz-plane (Figures 3E,F), and the main beam in the xy-plane is shifted away from the target by about 0.5 mm (Figure 3G). The experimental results were in good agreement with the simulations. The ultrasound field generated by the BAM with skull aberration correction is shown in Figures 3H–J. Correction enabled precisely targeting. The respective FLHM and FWHM were 13.5 and 3.3 mm from simulations with correction, and 16.3 and 3.7 mm from experiments. The focal properties (FLHM and FWHM) of tFUS beam after aberration correction were comparable to those measured in free field.

### Binary acoustic metasurface for dynamic focusing

**FIGURE 4:**
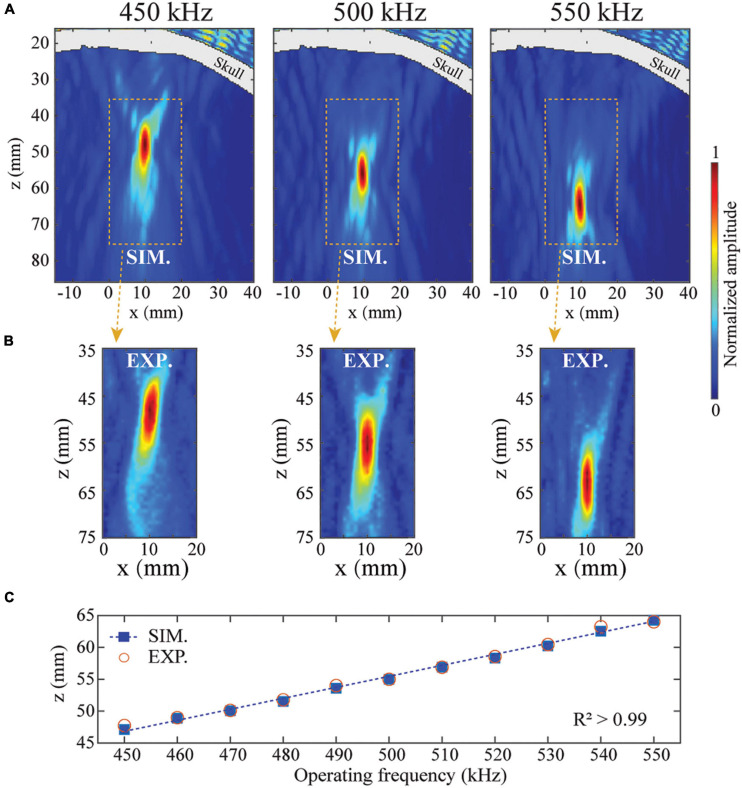
Dynamic steering of the tFUS focus. The transcranial focusing depth of BAM was electronically steered by changing the operating frequency of the ultrasound transducer. **(A,B)** Simulated and experimentally measured ultrasound fields in the xz-plane at 450, 500, and 550 kHz, respectively. **(C)** Simulated (solid squares) and experimentally measured (open circles) focal depths from 450 to 550 kHz.

After demonstrating the application of BAMs in skull aberration correction, we evaluated the property of BAMs in dynamic focusing. We evaluated the continuously dynamic focusing property of BAMs with correction for transcranial focusing by changing the operating frequency of the ultrasound transducer. As shown in Figures 4A,B, the focal depth of the BAM was dynamically tuned from 47.1 to 64.3 mm by increasing the operating frequency from 450 to 550 kHz. This range of dynamic focusing was limited by the bandwidth of the planar transducer. A linear relationship (*R*^2^ = 0.99) between the focal depth *z* and the operating frequency was found (Figure 4C). The experimental results agreed well with the numerical results. These data demonstrated that BAMs could electronically and continuously steer tFUS beam focus by adjusting the operating frequency of the ultrasound transducer.

### Binary acoustic metasurface for multi-point targeting

**FIGURE 5:**
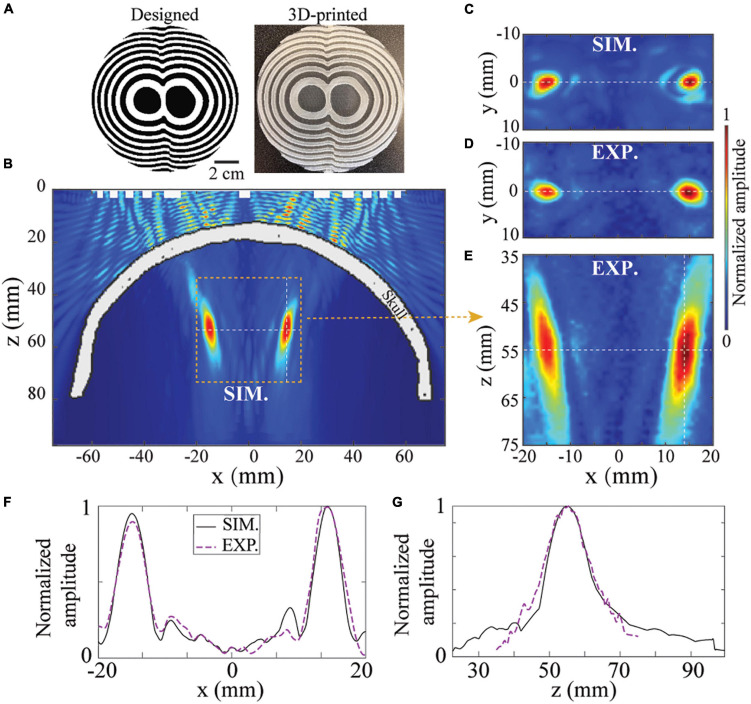
BAM for tFUS bifocal focusing. **(A)** The designed and 3D-printed BAM for bifocal focusing at *z* = 55mm. The ultrasound field in the xz-plane by simulation **(B)** and experiment **(E)**. **(C,D)** Corresponding xz-plane of ultrasound field. **(F,G)** Simulated and experimentally measured axial and lateral pressure profiles.

Besides single-point transcranial focusing, BAM can also be used for transcranial multi-point targeting. Figure 5A shows the designed and printed bifocal BAM for the bilateral focusing. It was designed with the bifocal points set to (x = −15,*y* = 0,*z* = 55)mm and (x=15,*y* = 0,*z* = 55)mm, respectively. Figures 5B,E show the ultrasound fields generated in the xz-plane by simulation and experimental measurements. The lateral ultrasound fields in the xy-plane are shown in Figures 5C,D. Excellent agreement was observed between the simulations and experiments. The lateral and axial ultrasound fields across the focus are shown in Figures 5F,G. The FLHM and FWHM from the experiments (18.5 and 5.4 mm) agree with the simulations (15.2 and 4.6 mm). These data demonstrated the capability of BAMs in transcranial multi-foci focusing.

## Discussion

This study designed and 3D printed BAMs for skull aberration correction and dynamic focusing of transcranial ultrasound. We numerically and experimentally validated the manufactured BAMs using an *ex vivo* human skullcap. Several features of the BAM were demonstrated. First, BAMs could correct ultrasound beam aberration induced by the skull and precise transcranial targeting was achieved with BAMs (Figure 3). Second, the focus of the BAM was steerable along the wave propagation direction (Figure 4) by adjusting the operating frequency of the incident wave. Third, multi-point focusing was achieved by BAMs, allowing simultaneous targeting of different locations (Figure 5).

Compared with the commonly used phased array technique, the proposed BAMs has several unique advantages. Phased arrays have the flexibility to achieve multiple functionalities, such as beam aberration correction, multi-point focusing, and beam steering; however, their high cost, bulky size and complex electronics hinder their broad applications. The 3D-printed BAMs can also achieve beam aberration correction, multipoint sonication, and dynamic focusing along the wave propagation direction. Moreover, they are low cost, easy to fabric, and do not require complex hardware. BAMs can potentially lower the barrier to the broad applications of tFUS by providing an affordable, simple-to-design and easy-to-use ultrasound device for customized usages. The proposed BAMs are not going to replace phased arrays when there is a need to achieve dynamic beam steering in 3D space; however, BAMs enables flexibility beamforming with the capability to form complex beam pattern and dynamically steer the beam pattern along the wave propagation direction.

This study has several limitations. First, the focusing properties (FLHM and FWHM) of BAMs are highly related to the acoustic properties of the 3D printing material. This study only tested PLA material. Future work is needed to investigate the performance of AB-BAMs printed with other materials. Second, this study only presented transcranial single and double focusing using BAMs. More complex beam patterns can be generated by binary lens as reported before (Ma et al., 2020). Future studies are needed to demonstrate the use of BAMs to generate complex beam patterns. Third, this study focused on the development of the tFUS devices. Future studies will explore the applications of these devices in different applications, such as neuromodulation and brain drug delivery.

## Conclusion

We designed and fabricated BAMs for tFUS. The BAMs were capable of correcting the beam aberration induced by the skull and achieving dynamic focusing. Numerical simulation and experimental studies using an *ex vivo* human skullcap validating their functionalities. We also demonstrated the application of the BAMs in generating multi-point focusing. The BAMs enables the development of affordable, simple-to-design and easy-to-use tFUS devices.

## Data availability statement

The original contributions presented in this study are included in the article/supplementary material, further inquiries can be directed to the corresponding author.

## Author contributions

ZH conceived the idea of the study. ZH, YY, LX, YH, and HC did experimental validation, analyzed the data, interpreted the results, and wrote the manuscript. HC and ZH contributed to critical writing and revision of the manuscript. All authors contributed to the article and approved the submitted version.
